# Endobronchial Epstein-Barr Virus Associated Post-transplant Lymphoproliferative Disorder in Hematopoietic Stem Cell Transplantation

**DOI:** 10.4137/ccrep.s2084

**Published:** 2009-02-03

**Authors:** S. Feuillet, V. Meignin, J. Brière, P. Brice, V. Rocha, G. Socié, A. Tazi, A. Bergeron

**Affiliations:** 1 Université Denis Diderot—Paris 7, Assistance Publique-Hôpitaux de Paris, Service de Pneumologie, Hôpital Saint-Louis, Paris, France.; 2 Université Denis Diderot—Paris 7, Assistance Publique-Hôpitaux de Paris, Service de Pathologie, Hôpital Saint-Louis, Paris, France.; 3 Université Denis Diderot—Paris 7, Assistance Publique-Hôpitaux de Paris, Service d’Hématologie, Hôpital Saint-Louis, Paris, France.; 4 Université Denis Diderot—Paris 7, Assistance Publique-Hôpitaux de Paris, Service de Greffe de moelle, Hôpital Saint-Louis, Paris, France.

**Keywords:** bronchoscopy, endobronchial patterns, epstein-barr virus, hematopoietic stem cell transplantation, post transplant lymphoproliferative disorders

## Abstract

The Epstein-Barr virus (EBV) associated Post-Transplant Lymphoproliferative Disorders (PTLD) are increasingly recognized as a fatal complication of hematological stem cell transplantation (HSCT). Thoracic involvement, that may be isolated or part of a disseminated disease, usually encompasses pulmonary nodules or masses and mediastinal lymph node enlargement.

The current case study presents 2 patients who underwent HSCT, one allogenic and the other autologous, who developed an exceptional endobronchial EBV related PTLD. The first patient had a fleshy white endobronchial mass resulting in a right upper lobe atelectasis and the second had an extensive necrotising mucosa from trachea to both basal bronchi without any significant change of lung parenchyma on the CT scan. In both cases, the diagnosis was made by bronchial biopsies. Physicians should be aware of an endobronchial pattern of EBV associated PTLD after HSCT to permit quick diagnosis and therapeutic intervention.

## Introduction

Post-transplant lymphoproliferative disorders (PTLD) are rare and life-threatening complications of prolonged immunosuppression that is usually derives from the proliferation of Epstein-Barr virus (EBV) infected B-cells.^1^ Although PTLD mainly occur in solid organ recipients, especially after lung transplantation, they also represent a life-threatening event complicating both allogenic and autologous bone marrow transplantation.^2^–^5^ PTLD can affect almost any organ or can be disseminated.^4^ Reported thoracic presentations of PTLD include solitary or multiple nodules or masses and/or mediastinal or hilar lymph nodes.^6^–^8^ In all these cases, the diagnosis of PTLD was made on lung biopsies, either CT scan guided or surgical.

It is reported here, an exceptional presentation of thoracic EBV related PTLD with endobronchial localization occurring in 2 hematological stem cell transplant recipients whose diagnosis was made by flexible bronchoscopy.

### Case 1

A-39-year old man underwent a bone marrow transplantation from an unrelated donor with 1/10 HLA-antigen mismatched for a chronic myeloid leukaemia in blastic phase, in September 2004. Pre-transplant EBV serological status was positive for both the patient and the donor. Because of graft failure, he subsequently received double cord blood stem cell transplantation on 4/11/2004, after fludarabine, cyclophosphamide and thymoglobuline as conditioning. Methotrexate and cyclosporine were given for graft-versus-host disease (GVHD) prophylaxis and valaciclovir for antiviral prophylaxis. While engraftment was successful, he experienced no GVHD in the post transplant course. Systematic quantitative EBV PCR performed for blood samples became positive 34 days after cord blood stem cell transplantation (65000 copies/mL). At this time, the patient had a normal lung CT scan. He was still receiving steroids 50 mg/d and cyclosporine 250 mg/d. As the result of a post transplant EBV reactivation, rituximab (administered at a dose of 375 mg/m^2^) was introduced with 4 subsequent weekly courses until December 2004, when PCR analysis of blood samples for EBV were significantly decreased (7500 copies/mL).

**Figure 1:**
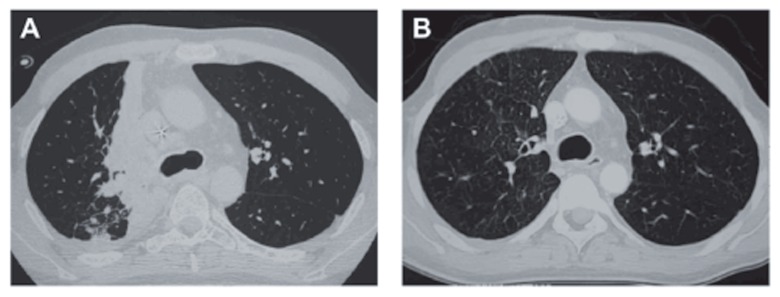
Evolution of lung computed tomography scan from patient 1. **A**) Eighty four days after cord blood transplantation, the scan showed a right hilar mass together with an atelectasis of the right upper lobe leading to the diagnosis of endobronchial post transplant lymphoproliferative disorder. **B**) Five months later, while the patient had received 3 courses of rituximab and cytotoxic chemotherapy, the scan was normalized.

**Figure 2:**
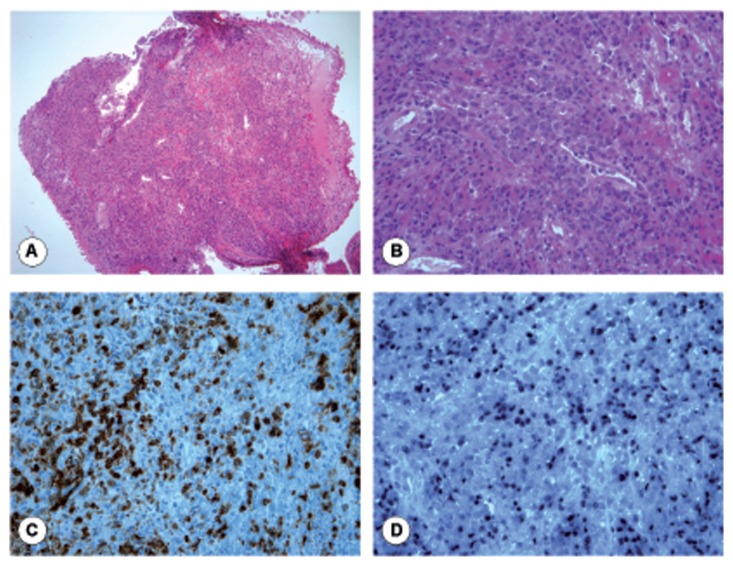
Histological findings of a bronchial biopsy from patient 1: **A**) and **B**) The bronchial lamina propria is infiltrated by polymorphic tumoral lymphoid cells (Hematoxylin eosin safran × 100, × 400). **C**) Immunohistochemical study shows a positive reaction for the B cell marker CD79a in the tumoral cells (× 400). **D**) In situ hybridization with EBERs probes shows that virtually all the tumoral cells are infected by the Epstein Barr virus (× 400).

He was hospitalised in January 2005 for a fever associated with a productive cough while he was still taking cyclosporine (250 mg/d) and prednisone (50 mg/d) as immunosuppressive treatment. No clinical signs of GVHD were noted. The chest radiograph and the CT scan showed a right hilar mass with atelectasis of the right upper bronchus (Fig. 1). There were no mediastinal or abdominal lymph nodes and the brain CT scan was normal. Bronchoscopy showed fleshy, white tissue, obstructing the origin of the right upper bronchus. The pathological findings of the biopsies of this lesion revealed a polymorphic lymphoproliferation expressing CD79A, CD30 and LMP1 (latent membrane protein 1 of the EBV). In situ hybridization of EBV-encoded RNA (EBER) was positive (Fig. 2). At this time, quantitative PCR for EBV was high both in blood sample and in the bronchial biopsy (20271 copies/mL and 23099 copies/mL respectively). The diagnosis of endobronchial EBV associated PTLD was retained and both cyclosporine and prednisone were stopped. The patient received 3 courses of rituximab (at the dose of 375 mg/m^2^), together with cyclophosphamide, doxorubicine, and vincristine. The right apical pulmonary abnormalities resolved both on the lung CT scan and bronchoscopy (Fig. 1). In June 2005, bronchial biopsies only showed scarring tissue and the PCR for EBV was negative both in blood and bronchial tissue samples. However, 3 months later, the patient had seizures that resulted from a left parietal abscess on brain MRI. Stereotaxic biopsy of this lesion confirmed a localization of EBV associated lymphoproliferation. At this time, quantitative PCR for EBV was still negative in the blood whereas it was slightly positive in the cerebrospinal fluid (143 copies/mL). After treatment with intrathecal rituximab and methotrexate, together with intravenous high dose methotrexate, the patient has been in complete remission until now.

### Case 2

A 67-year old man was treated with cyclophosphamide, etoposide, doxorubicin, prednisone and interferon α for a stage 4 follicular lymphoma from May 1998 until October 1999 with complete remission. In April 2002, a loss of weight, asthenia and abdominal pain were experienced by the patient. Complementary exams revealed abdominal and cervical lymph nodes as well as liver and spleen nodules. The diagnosis of a transformation to diffuse large B-cell lymphoma was made on the cytological analysis of a cervical adenopathy. A complete remission was obtained with 6 cycles of rituximab, etoposide, ifosfamide. He subsequently underwent autologous stem cell transplantation on January 2003 following BCNU, etoposide, cytarabine, melphalan as conditioning. Of note, EBV serological status was positive.

He relapsed in December 2004, with abdominal and inguinal adenopathies, as confirmed by the cytological analysis of an inguinal lymph node, characteristic of follicular lymphoma. He received no immunosuppressive treatment until August 2005, when he developed a productive cough and a dyspnoea together with a fever. The CT scans revealed necrotizing mediastinal lymphadenopathies without significant lung parenchymal opacities or abdominal lymph nodes. Bronchoscopy found an extensive necrosis from trachea to both basal bronchi. The pathological analysis of bronchial biopsies showed a polymorphic lymphoid infiltrate expressing CD20, CD30 and LMP1 of the EBV and a positive EBER. The EBV viral load in the blood and the bronchial biopsy samples evaluated by PCR was very high (27533 and 6107459 copies/mL respectively). The diagnosis of EBV associated PTLD was retained. The bacteriological findings of the bronchoalveolar lavage revealed *Staphylococcus Aureus*, *Escherichia coli* and *Proteus mirabilis*. Despite focussed antibiotics and one cycle of rituximab, the patient died a few weeks later.

## Discussion

As far as we know, these are the first ever reported cases of endobronchial EBV-associated lymphoproliferative disorders following hematopoietic stem cell transplantation.

Post transplant lymphoproliferative disorders (PTLD) encompass a heterogeneous group of abnormal lymphoid proliferations, mostly resulting from uncontrolled proliferation of EBV-transformed B-lymphocytes in the setting of profound immune dysfunction.^1^ Histologically, PTLD varies from a benign polyclonal lymphoid proliferation to a monoclonal proliferation with features of malignant lymphoma.^9^ EBV-associated PTLD have been most commonly described within the first year following solid organ transplantation predominantly after heart, lung or heart-lung transplantations.^10^,^11^ More rarely, PTLD may occur after hematological stem cell transplantation. After myeloablative allogenic stem cell transplantation, mainly in recipients of T-cell-depleted, HLA mismatched or an unrelated graft, the incidence of PTLD is approximately 1%.^2^,^5^ Similarly, after autologous stem cell transplantation, EBV-associated PTLD is a rare event described only in few scattered cases and is usually related to chemotherapy-induced immunosuppression.^3^,^4^,^12^,^13^ Notably, in a recent cohort of 218 autologous stem cell transplantations, no EBV related PTLD was reported.^14^ Although rare, PTLD represent a significant cause of mortality in these populations of patients.^4^

PTLD may involve extravisceral lymphoid tissue, any organ including liver, spleen, pharynx, central nervous system or can be disseminated.^4^ Pulmonary involvement, mainly described in lung transplant recipients, occurs in approximately 20% of cases, either isolated or as part of disseminated disease.^4^ The most common radiologic findings are solitary or multiple nodules, masses, enlarged mediastinal lymph nodes or pleural effusion.^4^,^6^,^7^,^8^ Recently, an additional case of EBV associated PTLD following allogenic bone marrow transplantation that was presented as interstitial pneumonia was reported.^15^ In all cases, the diagnosis necessitated CT scan guided transthoracic needle, transbronchial or open lung biopsies.

No information concerning potential endobronchial involvement of PTLD in hematological stem cell transplantation is available. It is noteworthy that our 2 patients have been presented here with a productive cough that is an unusual symptom of PTLD and compatible with a bronchial disease, as well as the presence of a right upper lobe atelectasis in patient 1. Although, in the setting of HSCT, bronchoscopy is usually performed for microbiological exploration of fever, respiratory symptoms and/or radiological lung abnormalities, PTLD should be considered in the differential diagnosis.

Prognosis of PTLD post HSCT is very poor with a high mortality exceeding 90% in all reported series.^4^ Therefore, the therapeutic intervention needs to occur as soon as the diagnosis of PTLD is made. Because of the close association with the impairment of immune system, the mainstay of therapy for PTLD is reduction in immunosuppression. However, many patients require more extensive therapy. Since recently, the use of the humanized mouse anti-human CD20 antibody (rituximab) has been proposed as a safe first line treatment for PTLD in patients who fail or do not tolerate reduction in immunosuppression.^16^ Thus, cytotoxic chemotherapy which is associated with a high rate of severe infectious complications in this setting should be reserved for patients who fail rituximab.^16^,^17^ Cellular immunotherapy with administration of EBV-specific cytotoxic T lymphocytes has also been proposed.^18^ When possible, surgical resection can result in complete remission.^4^ Endobronchial therapies should be of particular interest in patients with an isolated endobronchial localization of PTLD, as suggested by a successful photodynamic therapy combined with rituximab in a lung transplant recipient.^19^ Despite these treatments, relapses of EBV-associated PTLD after initial complete remission had been described as for our first patient. The incidence of these relapses is unknown.^17^

## Conclusion

PTLD is an increasingly recognized complication of HSCT. An awareness of the different pulmonary patterns of PTLD, particularly endobronchial, in the setting of hematopoietic stem cell transplantation is necessary for physicians to make prompt diagnosis and thus allow early intervention to improve the prognosis of this devastating complication of HSCT.
